# Cardiovascular toxicities of androgen deprivation therapy in Asian men with localized prostate cancer after curative radiotherapy: a registry-based observational study

**DOI:** 10.1186/s40959-022-00131-4

**Published:** 2022-03-14

**Authors:** Youquan Li, Whee Sze Ong, Ma Than Than Shwe, Nelson Ling Fung Yit, Sheriff Zhan Hong Quek, Eric Pei Ping Pang, Wen Shen Looi, Wen Long Nei, Michael Lian Chek Wang, Melvin Lee Kiang Chua, Terence Wee Kiat Tan, Eu Tiong Chua, Choon Ta Ng, Jeffrey Kit Loong Tuan

**Affiliations:** 1 Department of Radiation Oncology, National Cancer Centre Singapore, 11 Hospital Crescent, Singapore, 169610 Singapore; 2 Duke-NUS Graduate Medical School, Singapore, Singapore; 3 Division of Clinical Trials and Epidemiological Sciences, National Cancer Centre Singapore, Singapore, Singapore; 4 Department of Cardiology, National Heart Centre Singapore, Singapore, Singapore

**Keywords:** Cardiovascular toxicities, Androgen deprivation therapy, Major adverse cardiovascular events, Gonadotrophin-releasing hormone agonist & antagonist, Registry-based study, Southeast Asian population

## Abstract

**Background:**

Androgen deprivation therapy (ADT) and radiotherapy (RT) are the mainstay treatment for localized prostate cancer and recurrence after surgery. Cardiovascular (CV) toxicity of ADT is increasingly recognized, and the risk relates to pre-existing risk factors and ADT modalities. Despite ethnic differences in the prevalence of CV risk factors and variations of CV mortality, data on ADT-related cardiotoxicities in the Asian population remain inconclusive. Our registry-based study investigated ADT-related major adverse cardiovascular events (MACE) after primary or salvage RT.

**Methods:**

Our study combined two prospectively established registry databases from National Cancer Center Singapore and National Heart Center Singapore. The primary endpoint is time to first MACE after treatment. MACE is defined as myocardial infarction, stroke, unstable angina, or cardiovascular death. Two types of propensity score adjustments, including ADT propensity score as a covariate in the multivariable regression model and propensity score weighting, were applied to balance baseline features and CV risk factors between RT alone and RT + ADT groups.

**Results:**

From 2000 to 2019, 1940 patients received either RT alone (*n* = 494) or RT + ADT (*n* = 1446) were included. After a median follow-up of 10 years (RT) and 7.2 years (RT+ ADT), the cumulative incidence of MACE at 1, 3 and 9 years was 1.2, 5 and 16.2% in RT group, and 1.1, 5.2 and 17.6% in RT + ADT group, respectively. There were no differences in the incidence of MACE between 2 groups (HR 1.01, 95% CI 0.78–1.30, *p* = 0.969). Pre-treatment CV risk factors were common (80%), and CV disease (15.9%) was the second leading cause of death after prostate cancer (21.1%). On univariate analysis, older age, Indians and Malays, pre-existing CV risk factors, and history of MACE were associated with higher MACE risk. After propensity score adjustments, there remained no significant differences in MACE risk between RT + ADT and RT group on multivariable analysis.

**Conclusions:**

In our registry-based study, ADT is not associated with increased risk of major cardiovascular events among Southeast Asian men with prostate cancer after curative radiotherapy.

**Supplementary Information:**

The online version contains supplementary material available at 10.1186/s40959-022-00131-4.

## Introduction

Since the effect of castration on serum phosphatases in prostate cancer patients was first reported in 1941, androgen-deprivation therapy (ADT) has become the mainstay treatment for advanced and metastatic prostate cancer. Combined with radiotherapy (RT), ADT has been shown in several randomized trials to improves survival in intermediate-and high-risk localized disease, as well as locally advanced and node-positive disease [1–6]. However, ADT also causes a wide range of metabolic side effects including obesity, insulin resistance, and lipid alterations that contribute to cardiovascular (CV) risks.

Patients received curative radiotherapy are often diagnosed at an advanced age, and preexisting CV risk factors are common. ADT is most commonly given in the form of gonadotrophin-releasing hormone (GnRH) agonist with radiotherapy. In several retrospective studies, GnRH agonist is associated with higher CV risk compared with orchiectomy and GnRH antagonists [7–9]. Recent HERO trial reported a significant 54% reduction in new cardiovascular events for the oral GnRH-antagonist compared with the GnRH-agonist in advanced prostate cancer [7].

Cardiovascular disease has become the leading cause of death in men with prostate cancer in the United States [10]. Cardiovascular disease and cancer are the first and second most common cause of death in Singapore and the leading contributors to the burden of disease. A growing body of evidence demonstrates racial and ethnic disparities in the prevalence of CV risk factors and variations of CV mortality in the general population [11]. Ethnicity also impacts prostate cancer-specific outcomes of men with metastatic hormone-sensitive prostate cancer [12]. The ethnic differences of prostate cancer and cardiovascular disease both could be attributed to distinct biological, social, and environmental factors. However, data on ADT-related CV toxicities in the Asian prostate cancer population remains scanty and inconclusive [13–15]. The current study used real-world registry data to assess the association of ADT and major CV events in the Southeast Asian prostate cancer population treated with curative radiotherapy.

## Methods

### Patients

Outcomes Research in DRO Cancer Care (“Outcomes Study”) (CIRB ref.: 2016/2020/B) is a prospectively established registry database at National Cancer Centre Singapore. Outcomes Study collects comprehensive real-world data (RWD) through Electronic Medical Record (EMR) and patient-reported outcomes (PRO) during cancer patients’ entire treatment and follow-up period. Singapore Cardiac Data Bank (SCDB) was established in 2000 as a national data bank of cardiovascular diseases and procedures (NIH: NCT03760705). SCDB modules include various intervention procedures and cardiac surgery [16, 17]. Individual data from two registry databases were combined via a unique national identification number.

The current study included Singapore male residents with non-metastatic prostate cancer who started treatment from January 2000 to September 2019. Initial data collection of medical history was made by the treating radiation oncologist at the first consultation. Physicians systemically assessed cardiovascular risk factors including Body Mass Index (BMI), smoking history, diabetes mellitus, hypercholesterolemia, hypertension, stroke, coronary artery disease, type of intervention or surgery for cardiac conditions, medications, and other significant cardiac histories through EMR and history taking. New cardiovascular events after treatment and cause of death were verified by board certified cardiologists.

The decision on ADT was made by oncologists according to D’Amico risk classification in localized disease or pathological findings and PSA after radical prostatectomy [1, 4, 18]. ADT included GnRH antagonists and GnRH agonists with or without short-term anti-androgen therapy for testosterone flare prevention. Men who underwent orchiectomy were excluded. Primary radiation regimens included conventional fractionation (74–78 Gy,1.8–2.0 Gy per fraction), moderate hypofractionation (60 Gy, 2.5–4.0 Gy per fraction) or ultra-hypofractionation (> 5.0 Gy per fraction). Conventional fractionation (66 Gy, 2.0 Gy per fraction) was given as adjuvant or salvage radiotherapy after radical prostatectomy.

### Outcome measure and assessments

The primary endpoint was time to first Major Adverse Cardiovascular Events (MACE) after the start of treatment for prostate cancer. MACE was defined as myocardial infarction, stroke, unstable angina requiring intervention, or cardiovascular death. The start date of treatment was defined as the start of ADT for patients who had received RT and ADT (RT + ADT) and the start of RT for RT only patients. Time to MACE was censored at the initiation of palliative systemic therapy (ADT, other hormonal agents, or chemotherapy) when patients developed recurrence or metastases. Alive or lost to follow-up patients without recurrence, metastases or MACE were censored at their date of the last follow-up.

### Statistical analysis

Baseline characteristics of the two treatment groups were compared using the Chi-square or Fisher’s exact test for categorical variables and Mann-Whitney U test for continuous variables. A competing risk approach was used to estimate the cumulative incidence of MACE as the cause of failure, with deaths due to non-cardiovascular causes as competing events. Comparison of cumulative incidence curves was made using the Gray’s test.

Univariate and multivariable Fine and Gray’s regression model was used to examine the association of ADT and other covariates with time to first MACE. Variables with univariate *p* < 0.05 were included in the multivariable model. Proportional hazard (PH) assumption was verified for each variable included in the multivariable model by including a time-by-variable interaction term for each variable.

Sensitivity analysis was performed to assess the impact of unbalanced baseline characteristics on the association of ADT with the risk of MACE via 2 types of propensity score adjustments. The first adjustment was made by including the probability of receiving ADT (p) as a covariate in the multivariate Fine and Gray’s regression model for time to first MACE. The second adjustment was made via propensity score weighting where each RT + ADT patient was assigned a weight of (1/p) and each RT only patient was assigned a weight of (1/1-p). The probability of receiving ADT was derived based on a logistic regression analysis with all baseline characteristics included as covariates in the model. Goodness of fit and discrimination ability of the fitted logistic model were assessed based on the Hosmer-Lemeshow test and area under the receiver operating characteristics curve (AUC), respectively. All statistical analyses were performed using Stata 15.0. Statistical significance was defined as 2-sided *p* < 0.05.

## Results

The study cohort consisted of 1940 patients was analyzed, of which 494 received RT only and 1446 received RT + ADT. The baseline characteristics were summarized in Table 1. Majority (*n* = 1702, 87.7%) received primary radiotherapy; 12.3% (*n* = 238) were treated as salvage or adjuvant radiation. Among the RT + ADT patients, 96.9% (*n* = 1401) received GnRH agonists (Goserelin, Leuprorelin & Triptorelin) and 61.5% (*n* = 889) received more than 6 months’ hormonal treatment.

**Table 1:** Baseline Demographic and Treatment Characteristics

	RT only (*N* = 494)		RT + ADT (*N* = 1446)		*p*
	No.	%	No.	%	
Age at start of treatment, years					
Median (range)	68 (49–94)		71 (46–89)		< 0.001
Below 70	274	55.5	650	45.0	< 0.001
70 & over	220	44.5	796	55.0	
Ethnic group					0.677
Chinese	447	90.5	1297	89.7	
Malays	17	3.4	66	4.6	
Indians	20	4.0	50	3.5	
Others	10	2.0	33	2.3	
ECOG performance status					0.024
0–1	493	99.8	1425	98.5	
2–4	1	0.2	21	1.5	
Gleason score					< 0.001
6 or less	241	48.8	177	12.2	
7	197	39.9	770	53.3	
8–10	39	7.9	485	33.5	
Missing	17	3.4	14	1.0	
ISUP grade					< 0.001
1	241	48.8	177	12.2	
2	136	27.5	451	31.2	
3	61	12.3	319	22.1	
4	23	4.7	207	14.3	
5	16	3.2	278	19.2	
Missing	17	3.4	14	1.0	
D’Amico risk classification					< 0.001
Low	174	35.2	49	3.4	
Intermediate	198	40.1	419	29.0	
High	107	21.7	973	67.3	
Missing	15	3.0	5	0.3	
Radiation intent					< 0.001
Definitive radiation	354	71.7	1348	93.2	
Salvage radiation	140	28.3	98	6.8	
ADT type^a^					NA
Agonist	NA	NA	1401	96.9	
Antagonist	NA	NA	45	3.1	
ADT duration					NA
4–6 months	NA	NA	557	38.5	
> 6 months	NA	NA	889	61.5	
Baseline CVD risk factor					0.010
At least one	386	78.1	1209	83.6	
None	83	16.8	196	13.6	
Missing	25	5.1	41	2.8	
Previous history of MACE					0.127
No	398	80.6	1113	77.0	
Yes	95	19.2	332	23.0	
Missing	1	0.2	1	0.1	

*ECOG* Eastern Cooperative Oncology Group, *ISUP* International Society of Urological Pathology, *CVD* Cardiovascular disease, *NA* Not applicable

^a^Agonist included goserelin, leuprorelin & triptorelin; antagonist included degarelix

Preexisting cardiovascular risk factors were common in our study population. Over 80% of the patients had at least one risk factor across the three main domains: lifestyle (such as smoking and obesity defined as BMI > 27.5), cardiovascular (such as diabetes, dyslipidemia, and hypertension), and history of MACE. CV risk factors were more common in RT + ADT patients (83.6% versus 78.1%, *p* = 0.010), on account of the higher percentage of patients in this group with hypertension, dyslipidemia and diabetes (Supplementary Table SA1). Statins were also more commonly taken by RT + ADT patients (42.8% versus 35.2%, *p* = 0.002) (Supplementary Table SA2). About 1 in 5 patients in both RT alone (19%) and RT + ADT group (23%) had a previous history of MACE.

The median follow-up duration was 10 years (95% CI, 9.2–10.7 years) for RT alone group and 7.2 years (95% CI, 6.9–7.5 years) for RT + ADT group. This could be attributed to the higher incidence of deaths over time within the RT + ADT patients. The risk of all causes mortality amongst RT + ADT group was 1.23 times (95% CI 0.99–1.52) that of RT alone group (Supplement Table SA6). The RT + ADT group had higher risk of prostate cancer deaths (Sub distribution HR [SHR] 1.14, 95% CI 0.72–1.80, *p* = 0.574), as well as other deaths not related to prostate cancer and CV diseases (SHR 1.26, 95% CI 0.96–1.66, *p* = 0.094) than RT alone group.

**Fig. 1:**
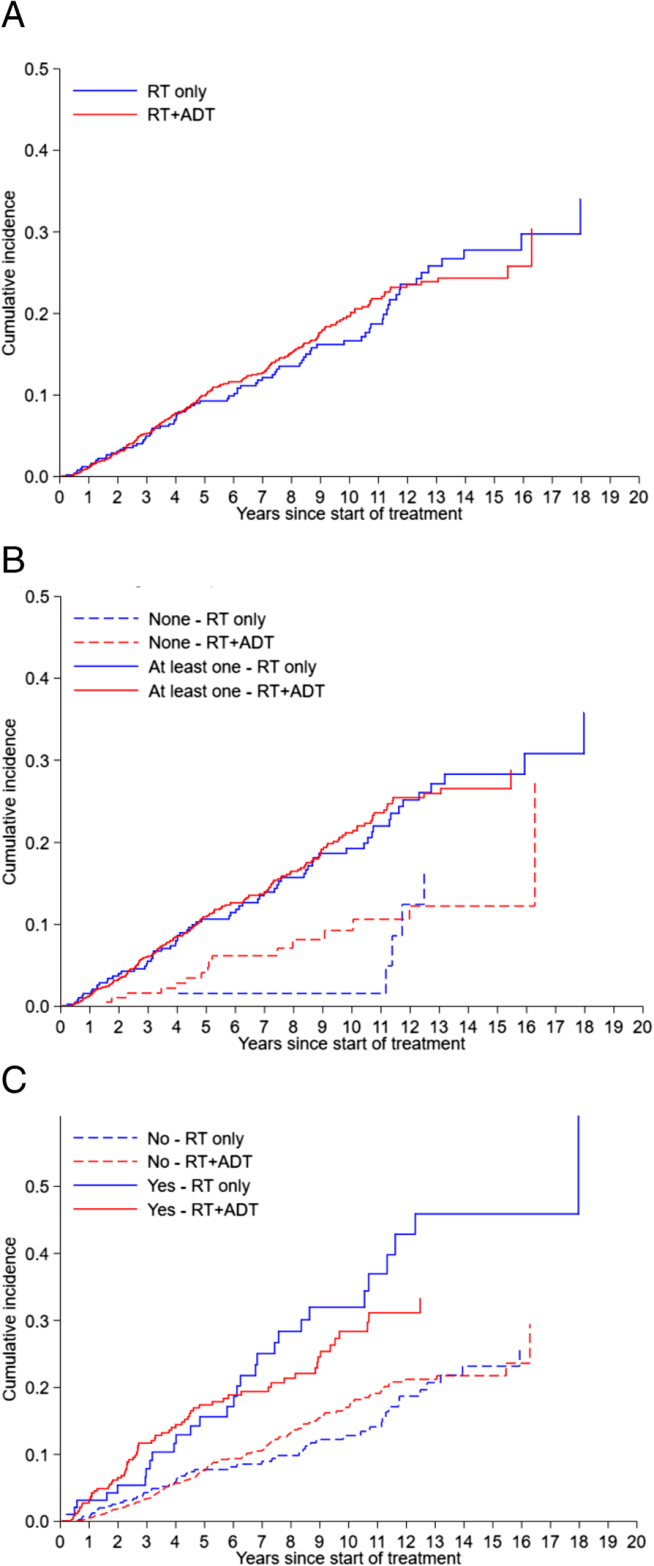
Cumulative incidence of MACE after start of treatment by treatment groups. **A**: Cumulative incidence of MACE in the entire cohort. (Subdistribution HR (RT only as reference) 1.01 (0.78–1.30), *p* = 0.969). **B**: Cumulative incidence of MACE by presence of baseline CVD risk factor. (Subdistribution HR (RT only as reference) None: 1.49 (0.56–3.97); At least one: 0.99 (0.75–1.31) Interaction’s *p* = 0.431). **C**: Cumulative incidence of MACE by presence of previous history of MACE. (Subdistribution HR (RT only as reference) No: 1.09 (0.79–1.50); Yes: 0.79 (0.51–1.21) Interaction’s *p* = 0.238)

The cumulative incidence of new MACE at 1, 3 and 9 years was 1.2, 5 and 16.2% in RT alone group, and 1.1, 5.2 and 17.6% in RT + ADT group, respectively (SHR 1.01, 95% CI 0.78–1.30, *p* = 0.969) (Fig. 1A). Myocardial infarction was the most common type of event in both groups (75% in RT and 66.5% in RT + ADT) (Supplement Table SA7). Subgroup analyses suggested that the risk of MACE by treatment groups did not vary by presence of baseline CV risk factor (None: SHR 1.49, 95% CI 0.56–3.97 vs At least one risk factor: SHR 0.99, 95% CI 0.75–1.31, interaction *p* = 0.431) (Fig. 1B) and history of MACE (No: SHR 1.09, 95% CI 0.79–1.50 vs Yes: SHR 0.79, 95% CI 0.51–1.21, interaction *p* = 0.238) (Fig. 1C). The cumulative incidence of MACE among patients with a history of MACE was numerically higher amongst the RT + ADT group than that of the RT alone group for the first few years after treatment, however, the difference was not statistically significant (*p* = 0.228). In particular, the incidence of new MACE at 1,2 and 3 years was 3.2, 5.4, and 7.9% in the RT group, and 3.0, 6.5, and 11.7% in the RT + ADT group, respectively. In terms of mortality, cardiovascular disease was the second most common cause of death (17.8%) after prostate cancer (21.1%) (Supplementary Table SA6).

On univariate analysis, older age (above 70), Malays and Indians (relative to Chinese), baseline CV risk factor and GnRH antagonist were associated with greater CV risk (Table 2). Patients with a history of MACE were also more likely to develop new MACE after treatment for prostate cancer (HR 2.05, 95% CI, 1.61–2.63; *p* < 0.001). Adjusting for all statistically significant factors in univariate analysis, there remained no significant differences in the risk of MACE between RT + ADT and RT groups on multivariable analysis.

**Table 2:** Univariate and multivariable analysis of MACE after start of treatment

	Univariate analysis		Multivariable analysis	
	Subdistribution HR (95% CI)	*p*	Subdistribution HR (95% CI)	*p*
Treatment group: RT + ADT vs RT only	1.01 (0.78–1.30)	0.969	0.95 (0.73–1.25)	0.722
Age (years): 70 & over vs below 70	1.41 (1.12–1.78)	0.004	1.35 (1.05–1.72)	0.018
Ethnic group: Malays vs Chinese	1.66 (1.04–2.65)	0.033	1.53 (0.93–2.51)	0.095
Ethnic group: Indians vs Chinese	2.15 (1.38–3.35)	0.001	1.79 (1.11–2.87)	0.016
Ethnic group: Others vs Chinese	1.37 (0.62–3.04)	0.436	1.57 (0.69–3.58)	0.282
Gleason score: 7 vs 6 or less	0.99 (0.75–1.30)	0.919		
Gleason score: 8–10 vs 6 or less	1.03 (0.74–1.43)	0.859		
ISUP grade: 2 vs 1	1.03 (0.76–1.40)	0.851		
ISUP grade: 3 vs 1	0.92 (0.64–1.31)	0.636		
ISUP grade: 4 vs 1	0.96 (0.61–1.50)	0.856		
ISUP grade: 5 vs 1	1.08 (0.74–1.56)	0.699		
D’Amico risk: Intermediate vs Low	0.87 (0.61–1.26)	0.472		
D’Amico risk: High vs Low	0.99 (0.71–1.39)	0.966		
Previous history of MACE: Yes vs No	2.05 (1.61–2.63)	< 0.001	1.75 (1.21–2.53)	0.003
Baseline CVD risk factor: ≥1 vs 0	2.53 (1.64–3.92)	< 0.001	1.73 (1.08–2.76)	0.022
Baseline metformin: Yes vs No	1.94 (1.46–2.56)	< 0.001	1.56 (1.15–2.10)	0.004
Baseline insulin: Yes vs No	3.10 (1.46–6.56)	0.003	1.53 (0.68–3.45)	0.310
Baseline statins: Yes vs No	1.61 (1.28–2.03)	< 0.001	1.21 (0.92–1.58)	0.174
Baseline antiplatelet: Yes vs No	1.61 (1.21–2.15)	0.001	0.78 (0.53–1.15)	0.213
Baseline anticoagulant: Yes vs No	1.82 (0.87–3.83)	0.113		
Surgery for IHD at baseline: Yes vs No	1.99 (1.47–2.71)	< 0.001	1.15 (0.76–1.75)	0.513
Radiation intent: Definitive vs Salvage	1.51 (0.98–2.32)	0.063		

*ISUP* International Society of Urological Pathology, *CVD* Cardiovascular disease, *IHD* Ischemic heart disease

In sensitivity analysis, the probability of receiving ADT was significantly associated with age, Gleason score, and D’Amico risk classification (Supplementary Table SA3). The fitted logistic model has a good fit (*p* = 0.409) and a high discrimination ability (AUC = 0.812). The conclusion that ADT was not associated with the risk of MACE from the main analysis remained unchanged in both propensity score adjustments (Supplementary Table SA4 and SA5).

## Discussion

In high-risk and locally advanced prostate cancer, ADT is an essential component combined with curative radiotherapy [2–4]. Several retrospective studies, mainly in the Caucasian population, suggested an increased risk of CV events in men who received GnRH agonist [7–9]. However, results of the CV toxicities of ADT in the Asian population are inconclusive. Teoh et al. reported ADT increased the risk of myocardial infarction and ischemic stroke compared with the non-ADT group in Chinese prostate cancer patients [13, 14]. However, a population study from Japan reported similar cardiovascular mortality after GnRH agonist compared with estimated mortality in the general population [15]. Another Taiwanese study reported similar incidence of coronary heart disease between men who did or did not receive ADT [19].

Cardiovascular disease is the leading cause of non-cancer deaths in men with prostate cancer. The incidence of secondary CV events after ADT varies greatly depending on underlying CV risk factors. A longitudinal study (RADICAL PC) reported that two-thirds of a cohort of 2492 men with prostate cancer had high CV risk with 22% having established CV disease [20]. In our cohort, 80% of men had at least one CV risk factor, and 20% had previous history of MACE. PRONOUNCE is the first prospective randomized study comparing CV safety of GnRH antagonist and agonist mainly in men with history of cardiac disorders. The study was terminated prematurely due to slow accrual, and no difference in MACE was observed at 1 year (5.5% vs 4.1%, *p* = 0.53) between degarelix or leuprolide groups [21]. In the HERO trial, new MACE within 1 year after ADT was common in patients with CV risk factors (6.2%) and a history of MACE (17.8%) [7]. GnRH antagonist (Relugolix) significantly reduced new MACE (2.9% for antagonist versus 6.2% for agonist) during the safety analysis, and the risk reduction was more significant in men with previous MACE (3.6% versus 17.8%) [7]. In our study, despite the preexisting CV risk factors are common (80%), new MACE within the first year was low; 1.1% for the entire group and 3.0% for patients with previous MACE. No difference of new MACE was found between radiation alone and radiation +ADT. Similar to previous studies from Japan and Taiwan populations [15, 19], ADT was not found to increase secondary CV events in our cohort.

The different findings on ADT-related cardiotoxicities between our cohort and previous studies in Caucasian population could be multi-factorial. First, identifying men at risk for secondary CV events after ADT remains challenging. The current approach mainly relies on routine CV risk factors assessment. Novel cardiac blood biomarkers including N-terminal pro-B-type natriuretic peptide (NTproBNP) C-reactive protein (CRP), and high-sensitivity troponin (hsTn) are being evaluated to predict ADT-related CV toxicities [22]. Novel predictive cardiac biomarkers are urgently needed to identify men at risk and mitigate ADT-related cardiotoxicities. Second, awareness of CV risk factors and secondary prevention with cardio-protective agents also impact the incidence of secondary MACE. The association between GnRH agonists and CV events has been hypothesized to relate to the destabilization of existing vascular plaques. The common usage of statins in our study (42.8% in RT + ADT and 35.2% in RT alone) might contribute to a favorable low incidence of new MACE [23–25]. In our study, the cardiac events were independently confirmed by cardiologists rather than insurance coding. The duration of ADT with radiation limits to 3 years instead of lifelong and this could explain the similar long-term CV events between two groups. Last, androgen receptor genetic polymorphisms and variation of androgen and sex hormone-binding globulin levels among different ethnic groups maybe another reason leading to differences in cardiovascular profiles and metabolic response to testosterone suppression [11, 26, 27]. In a population study from the United States, non-Hispanic Asians had the lowest risk of cardiac mortality among different racial groups [27].

Our observational study does have several limitations and needs interpretation within context. First, there is an inherent limitation for all retrospective studies, and the selection for ADT was not randomly assigned. It remains possible preexisting CV risk factors affect the decision on choice and duration of ADT. To minimize the selection bias, we performed a sensitivity analysis with propensity score adjustment for unbalanced baseline characteristics, and the conclusion remains the same. Second, despite initial cardiovascular risk assessment by oncologists, further management of various risk factors was done by the primary care physicians or cardiologists. However, prescription information within the EMR of the public healthcare system accurately determines the duration of ADT and other medications for primary or secondary prevention of cardiovascular disease. Third, Singapore Cardiac Databank includes information from the public healthcare system and may under-represented some of CV events from private providers. However, the database includes 80% of hospital care delivered in public institutions and reflects the nationwide pattern of practice. The strengths of the current study are the large size and complete long-term follow-up in the Southeast Asian men with prostate cancer from two prospectively established registry databases. Compared with previous studies of the Chinese or Japanese population, our study included a more diversified multi-ethnic group from Southeast Asia.

Cardiotoxicities associated with anticancer therapies are increasingly recognized and the prevention remains challenging in cancer survivorship [28]. Excessive CV morbidity and mortality associated with systemic therapy and radiotherapy compromised the clinical benefits of anticancer treatment [29]. Close collaboration among oncologists, cardiologists, and primary care physicians ensures delivery of optimal cancer care without compromising CV health [28, 30, 31].

## Conclusions

In our registry-based observational study, ADT was not found to be associated with increased risk of major cardiovascular event among Southeast Asian men received curative radiotherapy in two types of propensity score adjustments.

## Supplementary Information

**Additional file 1: Table SA1.** Baseline cardiovascular disease risk factor by treatment group. **Table SA2.** Baseline drug use and surgery/intervention for IHD by treatment group. **Table SA3.** Multivariable logistic regression of receiving RT + ADT treatment. **Table SA4.** Univariate and multivariable analysis of MACE after start of treatment with treatment propensity score included as a covariate. **Table SA5.** Univariate and multivariable analysis of MACE after start of treatment with treatment propensity score weighting. **Table SA6.** Cumulative Incidence Rate of Mortality by Causes. **Table SA7.** MACE by Type of Event.

## Data Availability

The datasets used and/or analysed during the current study are available from the corresponding author on reasonable request.
