# Supplementary material for: Cardiovascular toxicities of androgen deprivation therapy in Asian men with localized prostate cancer after curative radiotherapy: a registry-based observational study
Source: Cardiooncology. 2022 Mar 14;8:4. doi: 10.1186/s40959-022-00131-4 (PMC8919574; doi:10.1186/s40959-022-00131-4)
Supplement: Supplementary file 1 — Additional file 1: Table SA1. Baseline cardiovascular disease risk factor by treatment group. Table SA2. Baseline drug use and surgery/intervention for IHD by treatment group. Table SA3. Multivariable logistic regression of receiving RT + ADT treatment. Table SA4. Univariate and multivariable analysis of MACE after start of treatment with treatment propensity score included as a covariate. Table SA5. Univariate and multivariable analysis of MACE after start of treatment with treatment propensity score weighting. Table SA6. Cumulative Incidence Rate of Mortality by Causes. Table SA7. MACE by Type of Event. [file 40959_2022_131_MOESM1_ESM.docx]

Table SA1. Baseline cardiovascular disease risk factor by treatment group

|  | RT only (N=494) | | RT+ADT (N=1446) | | p |
| --- | --- | --- | --- | --- | --- |
|  | No. | % | No. | % |  |
| Baseline CVD risk factor |  |  |  |  | 0.010 |
| At least one | 386 | 78.1 | 1209 | 83.6 |  |
| None | 83 | 16.8 | 196 | 13.6 |  |
| Missing | 25 | 5.1 | 41 | 2.8 |  |
|  |  |  |  |  |  |
| Lifestyle factor |  |  |  |  |  |
| Smoking history |  |  |  |  | 0.264 |
| Current / former smoker | 137 | 27.7 | 449 | 31.1 |  |
| Never smoker | 334 | 67.6 | 944 | 65.3 |  |
| Missing | 23 | 4.7 | 53 | 3.7 |  |
|  |  |  |  |  |  |
| Alcohol history |  |  |  |  | 0.092 |
| Current drinker | 41 | 8.3 | 168 | 11.6 |  |
| Former drinker / Non-drinker^ | 424 | 85.8 | 1207 | 83.5 |  |
| Missing | 29 | 5.9 | 71 | 4.9 |  |
|  |  |  |  |  |  |
| Body Mass Index |  |  |  |  | <0.001 |
| 27.5 and above# | 65 | 13.2 | 242 | 16.7 |  |
| Below 27.5 | 347 | 70.2 | 1090 | 75.4 |  |
| Missing | 82 | 16.6 | 114 | 7.9 |  |
|  |  |  |  |  |  |
| Myocardial infraction / stroke history |  |  |  |  |  |
| Had myocardial infarction |  |  |  |  | 0.586 |
| Yes | 17 | 3.4 | 54 | 3.7 |  |
| No | 476 | 96.4 | 1391 | 96.2 |  |
| Missing | 1 | 0.2 | 1 | 0.1 |  |
|  |  |  |  |  |  |
| Had stroke |  |  |  |  | 0.605 |
| Yes | 21 | 4.3 | 59 | 4.1 |  |
| No | 472 | 95.5 | 1386 | 95.9 |  |
| Missing | 1 | 0.2 | 1 | 0.1 |  |
|  |  |  |  |  |  |
| Cardiovascular / cerebrovascular factor |  |  |  |  |  |
| Had hypertension |  |  |  |  | 0.002 |
| Yes | 265 | 53.6 | 896 | 62.0 |  |
| No | 228 | 46.2 | 549 | 38.0 |  |
| Missing | 1 | 0.2 | 1 | 0.1 |  |
|  |  |  |  |  |  |
| Had dyslipidemia |  |  |  |  | 0.001 |
| Yes | 193 | 39.1 | 690 | 47.7 |  |
| No | 299 | 60.5 | 755 | 52.2 |  |
| Missing | 2 | 0.4 | 1 | 0.1 |  |
|  |  |  |  |  |  |
| Had diabetes |  |  |  |  | 0.003 |
| Yes | 99 | 20.0 | 389 | 26.9 |  |
| No | 395 | 80.0 | 1056 | 73.0 |  |
| Missing | 0 | - | 1 | 0.1 |  |

CVD, cardiovascular disease

^Include social-drinker

#Asian body mass index’s cut-off for high risk of a range of non-communicable diseases

Table SA1. Baseline cardiovascular disease risk factor by treatment group (cont’d)

|  | RT only (N=494) | | RT+ADT (N=1446) | | p |
| --- | --- | --- | --- | --- | --- |
|  | No. | % | No. | % |  |
| Had angina |  |  |  |  | 0.010 |
| Yes | 9 | 1.8 | 63 | 4.4 |  |
| No | 484 | 98.0 | 1382 | 95.6 |  |
| Missing | 1 | 0.2 | 1 | 0.1 |  |
|  |  |  |  |  |  |
| Had heart failure |  |  |  |  | 0.163 |
| Yes | 0 | - | 8 | 0.6 |  |
| No | 493 | 99.8 | 1437 | 99.4 |  |
| Missing | 1 | 0.2 | 1 | 0.1 |  |
|  |  |  |  |  |  |
| Had atrial fibrillation /  cardiac dysrhythmia |  |  |  |  | 0.673 |
| Yes | 18 | 3.6 | 55 | 3.8 |  |
| No | 475 | 96.2 | 1390 | 96.1 |  |
| Missing | 1 | 0.2 | 1 | 0.1 |  |
|  |  |  |  |  |  |
| Had aneurysm |  |  |  |  | 0.373 |
| Yes | 0 | - | 3 | 0.2 |  |
| No | 493 | 99.8 | 1442 | 99.7 |  |
| Missing | 1 | 0.2 | 1 | 0.1 |  |
|  |  |  |  |  |  |
| Had coronary artery disease/  ischemic heart disease^ |  |  |  |  | 0.122 |
| Yes | 80 | 16.2 | 287 | 19.8 |  |
| No | 413 | 83.6 | 1158 | 80.1 |  |
| Missing | 1 | 0.2 | 1 | 0.1 |  |
|  |  |  |  |  |  |
| Had transient ischemic attack |  |  |  |  | 0.620 |
| Yes | 3 | 0.6 | 9 | 0.6 |  |
| No | 490 | 99.2 | 1436 | 99.3 |  |
| Missing | 1 | 0.2 | 1 | 0.1 |  |
|  |  |  |  |  |  |
| Had venous thromboembolism |  |  |  |  | 0.253 |
| Yes | 0 | - | 5 | 0.3 |  |
| No | 493 | 99.8 | 1440 | 99.6 |  |
| Missing | 1 | 0.2 | 1 | 0.1 |  |

^Include coronary heart disease

Table SA2. Baseline drug use and surgery/intervention for IHD by treatment group

|  | RT only (N=494) | | RT+ADT (N=1446) | | p |
| --- | --- | --- | --- | --- | --- |
|  | No. | % | No. | % |  |
| Had metformin |  |  |  |  | 0.551 |
| Yes | 68 | 13.8 | 227 | 15.7 |  |
| No | 424 | 85.8 | 1212 | 83.8 |  |
| Missing | 2 | 0.4 | 7 | 0.5 |  |
|  |  |  |  |  |  |
| Had insulin |  |  |  |  | 0.391 |
| Yes | 2 | 0.4 | 15 | 1.0 |  |
| No | 491 | 99.4 | 1424 | 98.5 |  |
| Missing | 1 | 0.2 | 7 | 0.5 |  |
|  |  |  |  |  |  |
| Had statins |  |  |  |  | 0.002 |
| Yes | 174 | 35.2 | 619 | 42.8 |  |
| No | 318 | 64.4 | 808 | 55.9 |  |
| Missing | 2 | 0.4 | 19 | 1.3 |  |
|  |  |  |  |  |  |
| Had antiplatelet |  |  |  |  | 0.001 |
| Yes | 58 | 11.7 | 251 | 17.4 |  |
| No | 431 | 87.2 | 1192 | 82.4 |  |
| Missing | 5 | 1.0 | 3 | 0.2 |  |
|  |  |  |  |  |  |
| Had anticoagulant |  |  |  |  | 0.969 |
| Yes | 9 | 1.8 | 25 | 1.7 |  |
| No | 480 | 97.2 | 1405 | 97.2 |  |
| Missing | 5 | 1.0 | 16 | 1.1 |  |
|  |  |  |  |  |  |
| Had surgery^ for coronary artery disease/ ischemic heart disease |  |  |  |  | 0.147 |
| Yes | 42 | 8.5 | 166 | 11.5 |  |
| No | 451 | 91.3 | 1277 | 88.3 |  |
| Missing | 1 | 0.2 | 3 | 0.2 |  |

^Coronary artery bypass grafting or percutaneous coronary intervention

Table SA3. Multivariable logistic regression of receiving RT+ADT treatment

|  | OR (95% CI) | p |
| --- | --- | --- |
| Age (years): 70 & over vs below 70 | 1.34 (1.03 - 1.73) | 0.027 |
|  |  |  |
| Ethnic group: Malays vs Chinese | 1.08 (0.55 - 2.12) | 0.829 |
| Ethnic group: Indians vs Chinese | 0.80 (0.42 - 1.53) | 0.496 |
| Ethnic group: Others vs Chinese | 1.20 (0.51 - 2.85) | 0.677 |
|  |  |  |
| Gleason score: 7 vs 6 or less | 2.70 (1.74 - 4.19) | <0.001 |
| Gleason score: 8-10 vs 6 or less | 4.71 (2.42 - 9.18) | <0.001 |
|  |  |  |
| ISUP grade: 2 vs 1 | 0.74 (0.51 - 1.06) | 0.108 |
| ISUP grade: 3 vs 1 | ^ | - |
| ISUP grade: 4 vs 1 | 0.48 (0.24 - 0.96) | 0.039 |
| ISUP grade: 5 vs 1 | ^ | - |
|  |  |  |
| D’Amico risk: Intermediate vs Low | 4.08 (2.52 - 6.59) | <0.001 |
| D’Amico risk: High vs Low | 14.67 (8.73 - 24.68) | <0.001 |
|  |  |  |
| Previous history of MACE: Yes vs No | 0.89 (0.55 - 1.45) | 0.650 |
|  |  |  |
| Baseline CVD risk factor: ≥1 vs 0 | 0.84 (0.57 - 1.23) | 0.365 |
|  |  |  |
| Baseline metformin: Yes vs No | 0.80 (0.56 - 1.15) | 0.235 |
|  |  |  |
| Baseline insulin: Yes vs No | 2.69 (0.48 - 15.10) | 0.261 |
|  |  |  |
| Baseline statins: Yes vs No | 1.28 (0.95 - 1.72) | 0.110 |
|  |  |  |
| Baseline antiplatelet: Yes vs No | 1.38 (0.85 - 2.25) | 0.197 |
|  |  |  |
| Baseline anticoagulant: Yes vs No | 0.94 (0.36 - 2.44) | 0.895 |
|  |  |  |
| Surgery for IHD at baseline: Yes vs No | 1.11 (0.63 - 1.94) | 0.720 |

ISUP, International Society of Urological Pathology; CVD, cardiovascular disease; IHD, ischemic heart disease

^Category omitted due to collinearity

Table SA4. Univariate and multivariable analysis of MACE after start of treatment with treatment propensity score included as a covariate

|  | Univariate analysis | |  | Multivariable analysis | |
| --- | --- | --- | --- | --- | --- |
|  | Subdistribution HR (95% CI) | p |  | Subdistribution HR (95% CI) | p |
| Treatment group: RT+ADT vs RT only | 1.01 (0.78 - 1.30) | 0.969 |  | 1.01 (0.73 - 1.39) | 0.957 |
|  |  |  |  |  |  |
| Age (years): 70 & over vs below 70) | 1.41 (1.12 - 1.78) | 0.004 |  | 1.39 (1.08 - 1.79) | 0.011 |
|  |  |  |  |  |  |
| Ethnic group: Malays vs Chinese | 1.66 (1.04 - 2.65) | 0.033 |  | 1.58 (0.96 - 2.61) | 0.070 |
| Ethnic group: Indians vs Chinese | 2.15 (1.38 - 3.35) | 0.001 |  | 1.83 (1.14 - 2.94) | 0.012 |
| Ethnic group: Others vs Chinese | 1.37 (0.62 - 3.04) | 0.436 |  | 1.57 (0.69 - 3.58) | 0.281 |
|  |  |  |  |  |  |
| Gleason score: 7 vs 6 or less | 0.99 (0.75 - 1.30) | 0.919 |  |  |  |
| Gleason score: 8-10 vs 6 or less | 1.03 (0.74 - 1.43) | 0.859 |  |  |  |
|  |  |  |  |  |  |
| ISUP grade: 2 vs 1 | 1.03 (0.76 - 1.40) | 0.851 |  |  |  |
| ISUP grade: 3 vs 1 | 0.92 (0.64 - 1.31) | 0.636 |  |  |  |
| ISUP grade: 4 vs 1 | 0.96 (0.61 - 1.50) | 0.856 |  |  |  |
| ISUP grade: 5 vs 1 | 1.08 (0.74 - 1.56) | 0.699 |  |  |  |
|  |  |  |  |  |  |
| D’Amico risk: Intermediate vs low | 0.87 (0.61 - 1.26) | 0.472 |  |  |  |
| D’Amico risk: High vs low | 0.99 (0.71 - 1.39) | 0.966 |  |  |  |
|  |  |  |  |  |  |
| Previous history of MACE: Yes vs No | 2.05 (1.61 - 2.63) | <0.001 |  | 1.75 (1.21 - 2.55) | 0.003 |
|  |  |  |  |  |  |
| Baseline CVD risk factor: ≥1 vs 0 | 2.53 (1.64 - 3.92) | <0.001 |  | 1.69 (1.06 - 2.70) | 0.028 |
|  |  |  |  |  |  |
| Baseline metformin: Yes vs No | 1.94 (1.46 - 2.56) | <0.001 |  | 1.60 (1.18 - 2.16) | 0.002 |
|  |  |  |  |  |  |
| Baseline insulin: Yes vs No | 3.10 (1.46 - 6.56) | 0.003 |  | 1.51 (0.66 - 3.42) | 0.328 |
|  |  |  |  |  |  |
| Baseline statins: Yes vs No | 1.61 (1.28 - 2.03) | <0.001 |  | 1.17 (0.88 - 1.54) | 0.276 |
|  |  |  |  |  |  |
| Baseline antiplatelet: Yes vs No | 1.61 (1.21 - 2.15) | 0.001 |  | 0.82 (0.55 - 1.23) | 0.335 |
|  |  |  |  |  |  |
| Baseline anticoagulant: Yes vs No | 1.82 (0.87 - 3.83) | 0.113 |  |  |  |
|  |  |  |  |  |  |
| Surgery for IHD at baseline: Yes vs No | 1.99 (1.47 - 2.71) | <0.001 |  | 1.14 (0.75 - 1.74) | 0.547 |
|  |  |  |  |  |  |
| Radiation intent: Definitive vs Salvage | 1.51 (0.98 - 2.32) | 0.063 |  |  |  |
|  |  |  |  |  |  |
| Propensity score (per 10% increase) | 1.01 (0.96 - 1.07) | 0.680 |  | 0.99 (0.93 - 1.05) | 0.676 |

ISUP, International Society of Urological Pathology; CVD, cardiovascular disease; IHD, ischemic heart disease

Table SA5. Univariate and multivariable analysis of MACE after start of treatment with treatment propensity score weighting

|  | Univariate analysis | |  | Multivariable analysis | |
| --- | --- | --- | --- | --- | --- |
|  | Subdistribution HR (95% CI) | p |  | Subdistribution HR (95% CI) | p |
| Treatment group: RT+ADT vs RT only | 0.94 (0.63 - 1.41) | 0.774 |  | 0.83 (0.54 - 1.28) | 0.397 |
|  |  |  |  |  |  |
| Age (years): 70 & over vs below 70) | 1.68 (1.14 – 2.48) | 0.008 |  | 1.36 (0.93 - 1.97) | 0.113 |
|  |  |  |  |  |  |
| Ethnic group: Malays vs Chinese | 1.44 (0.74 - 2.81) | 0.285 |  | 1.15 (0.54 - 2.46) | 0.715 |
| Ethnic group: Indians vs Chinese | 2.18 (1.00 - 4.74) | 0.049 |  | 1.67 (0.79 - 3.53) | 0.180 |
| Ethnic group: Others vs Chinese | 1.46 (0.54 - 3.91) | 0.456 |  | 1.78 (0.71 - 4.46) | 0.220 |
|  |  |  |  |  |  |
| Gleason score: 7 vs 6 or less | 0.98 (0.68 - 1.41) | 0.895 |  |  |  |
| Gleason score: 8-10 vs 6 or less | 1.42 (0.79 - 2.55) | 0.248 |  |  |  |
|  |  |  |  |  |  |
| ISUP grade: 2 vs 1 | 1.03 (0.71 - 1.51) | 0.862 |  |  |  |
| ISUP grade: 3 vs 1 | 0.89 (0.52 - 1.53) | 0.675 |  |  |  |
| ISUP grade: 4 vs 1 | 1.13 (0.54 - 2.38) | 0.746 |  |  |  |
| ISUP grade: 5 vs 1 | 1.62 (0.75 - 3.52) | 0.223 |  |  |  |
|  |  |  |  |  |  |
| D’Amico risk: Intermediate vs low | 1.01 (0.66 - 1.56) | 0.961 |  |  |  |
| D’Amico risk: High vs low | 1.18 (0.73 – 1.93) | 0.494 |  |  |  |
|  |  |  |  |  |  |
| Previous history of MACE: Yes vs No | 2.98 (1.98 - 4.49) | <0.001 |  | 2.15 (1.30 - 3.56) | 0.003 |
|  |  |  |  |  |  |
| Baseline CVD risk factor: ≥1 vs 0 | 3.43 (2.01 - 5.83) | <0.001 |  | 2.05 (1.08 - 3.89) | 0.028 |
|  |  |  |  |  |  |
| Baseline metformin: Yes vs No | 1.96 (1.14 - 3.36) | 0.015 |  | 1.55 (0.92 - 2.63) | 0.101 |
|  |  |  |  |  |  |
| Baseline insulin: Yes vs No | 2.18 (0.82 - 5.80) | 0.118 |  |  |  |
|  |  |  |  |  |  |
| Baseline statins: Yes vs No | 1.98 (1.32 - 2.97) | 0.001 |  | 1.28 (0.83 - 1.97) | 0.263 |
|  |  |  |  |  |  |
| Baseline antiplatelet: Yes vs No | 2.43 (1.46 - 4.05) | 0.001 |  | 1.04 (0.59 - 1.84) | 0.896 |
|  |  |  |  |  |  |
| Baseline anticoagulant: Yes vs No | 1.57 (0.56 - 4.37) | 0.392 |  |  |  |
|  |  |  |  |  |  |
| Surgery for IHD at baseline: Yes vs No | 2.44 (1.63 - 3.67) | <0.001 |  | 0.90 (0.45 - 1.82) | 0.779 |
|  |  |  |  |  |  |
| Radiation intent: Definitive vs Salvage | 2.23 (1.01 - 4.95) | 0.048 |  | 2.02 (0.94 - 4.33) | 0.071 |

ISUP, International Society of Urological Pathology; CVD, cardiovascular disease; IHD, ischemic heart disease

Table SA6. Cumulative Incidence Rate of Mortality by Causes

|  | All causes | | Prostate cancer | | CV disease | | Others | |
| --- | --- | --- | --- | --- | --- | --- | --- | --- |
|  | RT only | RT+ADT | RT only | RT+ADT | RT only | RT+ADT | RT only | RT+ADT |
| No. of patients | 494 | 1446 | 494 | 1446 | 494 | 1446 | 494 | 1446 |
| No. of events | 122 | 299 | 25 | 64 | 25 | 50 | 72 | 185 |
|  |  |  |  |  |  |  |  |  |
| CMIR, % |  |  |  |  |  |  |  |  |
| At 1 year | 1.2 | 0.6 | 0.6 | 0.1 | 0.2 | 0.1 | 0.4 | 0.3 |
| At 3 year | 5.4 | 4.2 | 1.0 | 0.8 | 1.3 | 1.1 | 3.0 | 2.3 |
| At 5 year | 9.6 | 11.0 | 1.8 | 2.6 | 2.5 | 2.4 | 5.3 | 5.9 |
| At 7 year | 14.9 | 15.8 | 2.7 | 3.5 | 3.4 | 3.1 | 8.8 | 9.1 |
| At 9 year | 19.8 | 24.0 | 3.3 | 5.4 | 5.4 | 4.2 | 11.1 | 14.6 |
| At 10 year | 23.7 | 29.1 | 4.1 | 6.4 | 5.4 | 4.7 | 14.2 | 18.0 |
|  |  |  |  |  |  |  |  |  |
| SHR (95% CI)^ | 1.23 (0.99 - 1.52) | | 1.14 (0.72 - 1.80) | | 0.86 (0.53 - 1.39) | | 1.26 (0.96 - 1.66) | |
| p# | 0.063 | | 0.574 | | 0.540 | | 0.094 | |

^Based on Fine and Gray’s regression model

#Based on Gray’s test

Table SA7. MACE by Type of Event

|  | RT only (N=494) | | RT+ADT (N=1446) | | p |
| --- | --- | --- | --- | --- | --- |
|  | No. | % | No. | % |  |
| No. of MACE | 80 | 100.0 | 206 | 100.0 | 0.440 |
| Myocardial infarction | 60 | 75.0 | 137 | 66.5 |  |
| Stroke | 10 | 12.5 | 37 | 18.0 |  |
| Unstable angina | 2 | 2.5 | 12 | 5.8 |  |
| CV death | 8 | 10.0 | 20 | 9.7 |  |
